# ^13^C NMR spectroscopy characterization of particle-size fractionated soil organic carbon in subalpine forest and grassland ecosystems

**DOI:** 10.1186/s40529-017-0179-5

**Published:** 2017-05-30

**Authors:** Yo-Jin Shiau, Jenn-Shing Chen, Tay-Lung Chung, Guanglong Tian, Chih-Yu Chiu

**Affiliations:** 1 Biodiversity Research Center, Academic Sinica, Nankang, Taipei, 11529 Taiwan; 2 Yung-Ta Institute of Technology and Commerce, Linluo, Pingdung, 90942 Taiwan; 3 Environmental Monitoring and Research Division, Monitoring and Research Department, Metropolitan Water Reclamation District of Greater Chicago (MWRD), Lue-Hing R&D Laboratory, 6001 W. Pershing Road, Cicero, IL 60804 USA

**Keywords:** ^13^C NMR, Particle size, Wildfire, Humification, Aromaticity, Soil organic carbon

## Abstract

**Background:**

Soil organic carbon (SOC) and carbon (C) functional groups in different particle-size fractions are important indicators of microbial activity and soil decomposition stages under wildfire disturbances. This research investigated a natural *Tsuga* forest and a nearby fire-induced grassland along a sampling transect in Central Taiwan with the aim to better understand the effect of forest wildfires on the change of SOC in different soil particle scales. Soil samples were separated into six particle sizes and SOC was characterized by solid-state ^13^C nuclear magnetic resonance spectroscopy in each fraction.

**Results:**

The SOC content was higher in forest than grassland soil in the particle-size fraction samples. The O-alkyl-C content (carbohydrate-derived structures) was higher in the grassland than the forest soils, but the alkyl-C content (recalcitrant substances) was higher in forest than grassland soils, for a higher humification degree (alkyl-C/O-alkyl-C ratio) in forest soils for all the soil particle-size fractions.

**Conclusions:**

High humification degree was found in forest soils. The similar aromaticity between forest and grassland soils might be attributed to the fire-induced aromatic-C content in the grassland that offsets the original difference between the forest and grassland. High alkyl-C content and humification degree and low C/N ratios in the fine particle-size fractions implied that undecomposed recalcitrant substances tended to accumulate in the fine fractions of soils.

## Background

Soil organic carbon (SOC) is one of the most important indicators of soil quality (Reeves 1997). It improves soil physical properties such as holding soil water and reducing soil bulk density (Manns and Berg 2014) and also helps in the development of the microbial community (Beyer 1995). The chemical composition of SOC in particle-size fractions may also affect soil microbial activity and decomposition rate of SOC (Beyer 1995). This information can be valuable for determining changes in the SOC pools with changes in plant cover or climate (Rossi et al. 2016).

Wildfire is one of the severe impacts that degrade SOC and alters vegetation (Fernandez et al. 2005) by affecting their content and composition (Czimczik et al. 2005; Knicker 2007). Labile carbon (C) compounds could be preferentially lost and lead to unalterable SOC during wildfire (Gonzalez-Perez et al. 2004) and further affect the physical, chemical, mineralogical, and biological properties of soil (Certini 2005; Shrestha and Chen 2010). The effect of wildfire on SOC content and properties depend on fire type (Mataix-Solera et al. 2011), vegetation (da Silva and Batalha 2008), climate (Birkeland 1999), and soil development (Certini 2005). Wildfire also decreases humic substance content and affects the aromaticity of humified fractions (Vergnoux et al. 2011b).

Solid-state ^13^C nuclear magnetic resonance spectroscopy with cross-polarization and magic-angle spinning (CP-MAS ^13^C NMR) has been found as a useful tool to determine the composition of SOC (Golchin et al. 1997; Faria et al. 2015). It is also useful to evaluate changes in SOC pools and humification under different environmental impacts. For example, Rossi et al. (2016) used ^13^C NMR spectroscopy to characterize the change in composition and structure of SOC after fire disturbance. Faria et al. (2015) used the NMR spectroscopy technique and found that wildfire increased the aromaticity of the topsoil SOC in forest in Portugal. Similarly, Lopez-Martin et al. (2016) measured the changes in SOC pools after wildfire burnt a mountain forest in Andalusia and found that fire-affected soils retained similar C and N content but showed higher aromaticity as compared with adjacent unburnt forest soils. However, previous research on composition of SOC pools of fire-affected soils were mostly based on whole bulk soils, and the composition of SOC in particle-size fractions may be worth studying.

High easily decomposable substances (O-alkyl-C) was found in the litter of grassland (i.e. bamboo), which may reduce the humification degree (alkyl-C/O-alkyl-C ratio) of the grassland soils (Wang et al. 2016). This easily decomposable litter may potentially remediate the impact of wildfire to the SOC pools and may also be worth studying.

The previous studies found higher amount of SOC as well as fungal and bacterial respiration rates in a *Tsuga* forest than a nearby fire-affected grassland (Imberger and Chiu 2001). As well, the diversity of the bacterial community was greater in the grassland than *Tsuga* forest soil (Lin et al. 2010). Microbial (fungi and bacteria) biomass appeared to be greater in large (>205 μm) than small particle-size fractions (Chiu et al. 2006). Moreover, the composition of SOC from the nearby forests showed greater humification degree in forest soil than dwarfed bamboo soil (Chen and Chiu 2003).

This research further determined the change in SOC in an original subalpine forest soil with vegetation succession after wildfire using a transect study. By determining the composition of SOC in various particle sizes along a sampling transect using ^13^C NMR spectroscopy, the study aims to better understand the effect of forest wildfires on the change of SOC in different soil particle scales. The hypothesis of this research is that higher humification degree and aromaticity would be found in the fine particle-size fractions as recalcitrant substances should accumulate in the fine fractions of soils.

## Methods

### Study site and sampling

Soil samples were collected from Tatajia, the saddle of Yushan, in central Taiwan (23°28′N, 120°54′E). The altitude of the study location was about 2700 m, with mean annual precipitation 4100 mm and temperature 9.5 °C. The study area geologically consisted of metamorphosed sedimentary rock (Miocene Epoch) comprising sandstone and shale.

Two distinct vegetation zones, coniferous forest and grassland, were visually identified in the study locations. Chinese hemlock (*Tsuga chinensis*) was the dominant species in the coniferous forest, and Taiwan red cypress (*Chamaecyparis formosensis*), Morrison spruce (*Picea morrisonicola*) and Armand’s pine (*Pinus armandi*) were found in the area. A grassland formed from a wildfire event approximately 50 years ago adjacent to the forest was used as a control, with dwarfed bamboo (*Yushania niitakayamensis*) and alpine silver grass (*Miscanthus transmorrisonensis*) the dominant plants. Between the grassland and forest was a transition zone inhabited by both bamboo and hemlock.

Soil was poorly developed due to the steep erosive terrain and classified as Typic Haplorthod in the forest, Typic Haplumbtept in the transition zone, and Umbric Dystrochrept in the grassland (Imberger and Chiu 2001).

After carefully removing the litter layer, one surface (0–10 cm) soil sample was collected from the center of each vegetation zone including forest, transition, and grassland by using a soil auger (8 cm diameter and 10 cm deep). Visible materials such as litter and roots were manually removed before sieving through a 2-mm sieve. The fresh soil samples were kept at 4 °C in the dark before analysis.

### Sample preparation and analyses

Part of the sieved soil samples (<2 mm) were air-dried and used for physical and chemical analysis. Soil texture (Gee and Bauder 1986), pH (McLean 1982), cation exchange capacity (Thomas 1982), and percentage of base saturation (Thomas 1982) were determined.

Other parts of sieved soil samples were treated with low-energy sonication and then separated to different particle-size fractions by a combination of wet sieving and continuous flow centrifugation (Oades et al. 1987). Six fractions, coarse (>250 μm), large (53–250 μm), medium (20–53 μm), small (2–20 μm), fine (2–0.4 μm), and very fine (<0.4 μm), were separated by sieving and centrifugation (Chiu et al. 2006). All fractionated samples were freeze-dried and stored for further analyses. Subsamples of each soil fractions were used to analyze total organic carbon (TOC) and total nitrogen (TN) using an elemental analyzer (NA1500 Series 2, Fisons, Italy).

Because of the complexity of separating soil samples to different particle-size fractions, only one set of soil particle-size fractions for each site was prepared for analyzing its ^13^C NMR.

### ^13^C NMR spectroscopy

The C functional groups of whole soils and particle-size fractions were examined by solid-state CP-MAS ^13^C NMR spectroscopy (Bruker DSX 400 MHz solid-state NMR, Germany). About 500 mg of the whole soils and particle-size fraction samples from each vegetation zone was used for each analysis. Acquisition conditions were spectrometer frequency, 100.46 MHz; spectra width, 20 kHz; spinning speed, 7 kHz; contact time, 6 ms; pulse delay time, 1 s; number of scans, 5000; spectra plotted region, 0–200 ppm.

Experimental procedures basically followed Jien et al. (2011). Briefly, the C functional groups were divided into 4 categories based on their chemical-shift areas: 0–50 ppm (alkyl-C), 50–110 ppm (O-alkyl-C), 110–165 ppm (aromatic-C), and 165–190 ppm (carboxyl-C). Each C functional group was determined by integrating the signal intensity of the designated spectrum ranges from ^13^C NMR. Aromaticity was calculated as the ratio of aromatic-C to the sum of alkyl-C, O-alkyl-C and aromatic-C (Hatcher et al. 1981).

### Statistical analysis

Because only one whole soil sample was obtained from each vegetation type, the degree of freedom of the data was not enough to process statistical analysis.

While for the particle size fractions, effects of difference of TOC, TN, C/N ratio, alkyl-C, O-alkyl-C, aromatic-C, carboxyl-C, aromaticity and humification degree on vegetation types and soil particle sizes were each analyzed by crossed two-way factorial analysis (3 vegetations × 6 particle sizes). Particle size was treated as a numerical variable and the average particle size was used to represent each fraction. When testing different variables on vegetations, different particle sizes were treated as random effect and vice versa. Tukey’s honestly significant difference (HSD) test and simple linear regression were respectively applied to further test the difference of each variable with different vegetations and with different particle sizes. Percentages of different C functional groups were compared with paired *t* test.

Because of the limited amounts of samples, *P* < 0.1 was considered statistically significant for all analyses.

## Results

### Characteristics of soil particle-size fractions

Selected physical and chemical properties of whole soils are in Table 1. The soil texture in this study site was sandy loam. The soils were strong acidic (pH 3.8–4.0), a typical soil characteristic in subalpine and alpine forest in Taiwan. Soil particle-size fractions in the 3 locations were mostly medium (20–53 μm) and large (53–250 μm), followed by coarse (>250 μm) (Table 2). Relatively low proportions (<15%) of soils were in fine (0.4–2 μm) and very fine (<0.4 μm) fraction.

**Table 1:** Soil physicochemical characteristics and particle-size distribution at 0–10 cm depth in different vegetation soils

Soil	Sand (%)	Silt (%)	Clay (%)	pH (H_2_O)	TOC^a^	TN^b^	C/N	CEC^c^ (cmole kg^−1^)	BS^d^ (%)
					(g kg^−1^)				
Forest	65.2	17.9	16.9	3.8	145.0	9.7	14.9	18.3	3.0
Transition	63.3	18.8	17.9	4.0	96.0	5.9	16.2	17.0	1.4
Grassland	46.0	27.6	26.4	3.8	97.0	5.6	17.4	16.7	2.9

^a^Total organic carbon

^b^Total nitrogen

^c^Cation exchangeable capacity

^d^Base saturation

**Table 2:** Particle-size distribution in different sampling locations

Location	% of particle size					
	Very fine (<0.4 μm)	Fine (0.4–2 μm)	Small (2–20 μm)	Medium (20–53 μm)	Large (53–250 μm)	Coarse (>250 μm)
Forest	1.3	4.1	7.8	33.1	34.5	19.2
Transition	3.3	6.8	8.1	29.7	31.8	20.3
Grassland	6.6	7.5	11.1	24.3	29.9	20.6

TOC and TN content in different particle-size fractions was seemingly increased with decreased particle sizes, but was masked statistically (P = 0.62 and 0.15, respectively) (Table 3). TOC content was higher in forest than in the grassland and transition zone (P < 0.001), with no difference in C/N ratio or TN among the three locations.

**Table 3:** Content of total organic C, N and C/N ratio in each particle-size fraction in different sampling locations

Site	Particle size (μm)	TOC^a^ (%)	TN^b^ (%)	C/N
Forest	Coarse (>250)	19.5	1.1	17.9
	Large (250–53)	15.7	1.0	16.4
	Medium (53–20)	19.8	1.3	15.6
	Small (20–2)	22.7	1.9	11.8
	Fine (2–0.4)	25.4	2.9	8.9
	Very fine (<0.4)	27.7	3.3	8.5
Transition	Coarse (<250)	12.4	0.6	21.8
	Large (250–53)	5.8	0.3	17.5
	Medium (53–20)	8.3	0.8	10.8
	Small (20–2)	11.2	0.8	14.1
	Fine (2–0.4)	10.2	0.9	11.8
	Very fine (<0.4)	12.5	1.1	11.4
Grassland	Coarse (>250)	15.6	1.0	15.3
	Large (250–53)	14.8	0.8	17.7
	Medium (53–20)	9.9	0.6	16.8
	Small (20–2)	19.1	1.5	12.6
	Fine (2–0.4)	18.8	3.2	5.8
	Very fine (<0.4)	22.3	2.3	9.9

^a^Total organic carbon

^b^Total nitrogen

The percentage content of C/N ratios at all locations basically decreased with decreasing particle size (*P* = 0.01).

### ^13^C NMR spectra in soil particle-size fractions

**Fig. 1:**
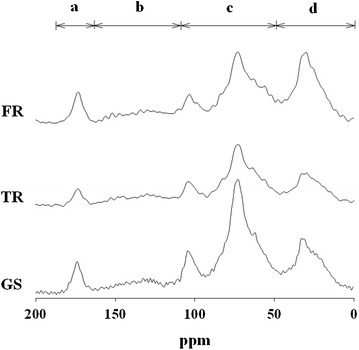
CP/MAS ^13^C NMR spectra of whole soils in the O/A horizon in forest (FR), transition (TR), and grassland (GS) sites. *a* carboxyl-C, *b* aromatic-C, *c* O-alkyl-C, *d* alkyl-C

**Fig. 2:**
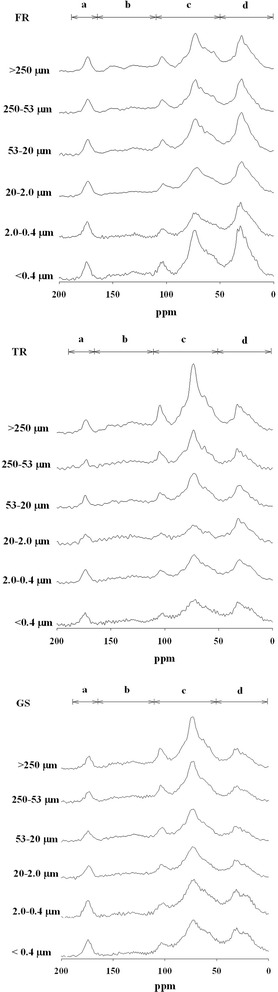
CP/MAS ^13^C NMR spectra in different particle-size fractions in forest (FR), transition (TR), and grassland (GS) sites. *a* Carboxyl-C, *b* aromatic-C, *c* O-alkyl-C, *d* alkyl-C

The integration ^13^C NMR provided the general composition of whole SOC in different vegetation zones (Table 4; Fig. 1). The proportion of different C fractions in different soil particle sizes is shown in Table 5. In comparing the C groups in different particle sizes in the three sampling zones, O-Alkyl (*P* = 0.007) was more abundant in large than small particles, whereas alkyl-C (*P* = 0.044) and carboxyl-C (*P* = 0.033) were more abundant in small than large particles (Fig. 2).

**Table 4:** CP/MAS ^13^C NMR of distribution of organic carbon functional group of whole soils in different sampling locations

Site	% of C functional groups				HI^a^	AR^b^
	Carboxyl-C	Aromatic-C	O-Alkyl-C	Alkyl-C		
Forest	7.42	12.29	51.18	29.11	0.70	0.13
Transition	7.82	14.84	49.11	26.82	0.68	0.16
Grassland	7.63	12.72	52.39	26.52	0.63	0.14

^a^Humification degree of sampling soils: alkyl-C peak area (0–50 ppm)/O-alkyl-C peak area (50–110 ppm)

^b^Aromaticity ratio: aromatic-C peak area (110–165 ppm)/total peak area (0–165 ppm)

**Table 5:** Relative intensities of CP/MAS ^13^C NMR spectra in different particle-size fractions in different sampling locations

Site	Particle size	% of C functional groups				HI^a^	AR^b^
	(μm)	Carboxyl	Aromatic	O-Alkyl	Alkyl		
Forest	Coarse (>250)	6.71	13.72	45.52	34.04	0.92	14.71
	Large (250–53)	7.31	14.55	44.77	33.37	0.93	15.70
	Medium (53–20)	6.35	13.20	43.43	37.02	1.06	14.10
	Small (20–2)	7.12	11.14	43.32	38.43	1.11	11.99
	Fine (2–0.4)	8.91	7.73	40.75	42.62	1.25	8.48
	Very fine (<0.4)	5.61	10.46	45.18	38.74	1.03	11.08
Transition	Coarse (>250)	5.13	16.25	55.04	23.58	0.53	17.13
	Large (250–53)	6.95	21.20	49.27	22.58	0.59	22.78
	Medium (53–20)	6.90	19.19	47.75	26.16	0.71	20.61
	Small (20–2)	7.57	22.77	36.88	32.79	1.22	24.63
	Fine (2–0.4)	7.45	16.93	44.16	31.45	0.92	18.29
	Very fine (<0.4)	7.48	14.90	44.50	33.11	0.95	16.11
Grassland	Coarse (>250)	6.03	14.95	56.14	22.88	0.51	15.91
	Large (250–53)	6.78	16.79	53.95	22.48	0.53	18.02
	Medium (53–20)	7.54	18.49	51.01	22.96	0.58	20.00
	Small (20–2)	7.66	15.25	51.12	25.96	0.65	16.52
	Fine (2–0.4)	6.65	8.10	51.76	33.50	0.80	8.68
	Very fine (<0.4)	6.66	7.50	50.22	35.62	0.87	8.03

^a^Humification degree of sampling soils: alkyl-C peak area (0–50 ppm)/O-alkyl-C peak area (50–110 ppm)

^b^Aromaticity ratio: aromatic-C peak area (110–165 ppm)/total peak area (0–165 ppm)

In addition, the O-alkyl-C content in all particle sizes was higher in grassland than forest (*P* = 0.005). Alkyl-C content was relatively high in forest soil samples (*P* = 0.004). Despite a slight difference in the distribution of C content under different vegetation, the transition zone and grassland showed similar trends in all different C functional groups except for aromatic-C, which was higher in the transition zone than the grassland soils (*P* = 0.01).

Interactions between vegetations and soil particle sizes all showed no significant difference in different C functional groups.

## Discussion

Wildfire rapidly oxidized organic matter at topsoil horizons and caused depletion of active C pool in an ecosystem (Gonzalez-Perez et al. 2004). Among the three sampling locations in present study, soil TOC appeared to be lower in burnt grassland and transition zones than in forest soils. In addition, Robichaud (2000) suggested that soil permeability and hydraulic conductivity were significantly decreased in fire-induced soil. This suggestion may explain the greater clay content in grassland than forest soil we found.

Distribution of C functional groups in fire-induced grassland soils showed similar patterns to that in forest soils. The major component of SOC in the three sampling locations was O-alkyl-C, which was mainly contributed by carbohydrate-derived structures.

The second greatest C functional group in the soil was alkyl-C, with higher content in forest than grassland soil (*P* = 0.002). The content is mostly from recalcitrant substances such as fatty acids and waxes (Mahieu et al. 1999). Jien et al. (2011) found coniferous vegetation with high content of alkyl-C, which was attributed to selective preservation of alkyl-C from lipids and aliphatic substances (Tegelaar et al. 1995).

Forest soils typically contain richer SOC and provide more aromatic-C and alkyl-C than grasslands because of the higher aromatic-C content (Golchin et al. 1997). By comparison, grassland (dwarfed bamboo) litter contains more O-alkyl-C, which can be more easily decomposed than that in coniferous forest (Wang et al. 2016). Moreover, previous research also showed that labile C can be significantly increased in the bamboo soil (Shiau et al. 2017; Wang et al. 2016). This fundamental difference in litter composition between the plants may remediate the wildfire affected SOC pools in the grassland. As the humification degree is calculated by alkyl-C/O-alkyl-C, higher O-alkyl-C provided by bamboo litter may result in the lower humification as we found in bamboo soils (*P* = 0.008). This observation also showed the impact of wildfire on the humification degree of SOC may recover after 50 years of succession.

The aromaticity (aromatic-C/total C functional groups ratio) was similar between the forest and grassland soils. Several studies found that wildfire and incomplete combustion increased the soil aromatic-C content and aromaticity (Vergnoux et al. 2011a; Faria et al. 2015; Rossi et al. 2016). However, aromatic-C is dominant in recalcitrant substances such as cutins, lignin, lipids, resins, surface waxes and tannin (Wang et al. 2016), and is usually found in woody forest soils. The potential increase in aromatic-C content via combustion in the studied grassland soil might offset the high aromatic-C content originally in the forest soils and litters, since little difference in aromaticity was observed between the forest and grassland soils.

Characterization of particle-size fractions is useful for process-oriented research into SOC (Mathers et al. 2000). Soluble organic C is readily utilized by soil microbes, whereas particulate SOC is a more important nutrient source for microbial activity (Mahieu et al. 1999; Chen and Chiu 2003). The results from all our sampling locations showed decreased O-alkyl-C peaks and increased alkyl-C peaks with decreasing soil particle size. This observation implied that the undecomposed recalcitrant substances tended to accumulate in the fine fractions of soils. The observation was also found in previous research in that the recalcitrant soil organic compounds were typically found stably binding with fine clay minerals (Calabi-Floody et al. 2012), whereas coarse particle-size fractions contained the major proportion of O-alkyl-C and aromatic-C materials (Kavdir et al. 2005). In addition, the finding of high alkyl-C spectra was consistent with low C/N ratios in the fine particle-size fractions, which suggests that the decomposition degree of organic materials was highest in the fine (<0.4 μm) particle fraction.

## Conclusions

Fire-affected grassland soil appeared to have lower TOC than forest soils in all soil particle-size fractions in our study. The humification degree was higher in forest than grassland soils. While the aromaticity was similar in forest and grassland soils, which might be attributed to the fire-induced aromatic-C content in the grassland that offsets the original difference in those characteristics between the forest and grassland.

The fine particle-size fraction contained a high amount of alkyl-C and high humification, which implied that the undecomposed recalcitrant substances tended to accumulate in fine particle-size soil fractions. In addition, the low C/N ratios of the fine particle-size fractions was supported with high alkyl-C in fine particles.

## Abbreviations

C: carbon
N: nitrogen
SOC: soil organic carbon
TOC: total organic carbon
TN: total nitrogen
NMR: nuclear magnetic resonance
CP-MAS: cross-polarization and magic-angle spinning
^13^C NMR: ^13^C nuclear magnetic resonance
